# Microstructures of Directionally Solidified Nb_15_Ti_55_Fe_30_ Alloy and Its Hydrogen Permeation Properties in the Presence of H_2_S

**DOI:** 10.3390/membranes14120253

**Published:** 2024-12-02

**Authors:** Erhu Yan, Guanzhong Huang, Kexiang Zhang, Lizhen Tao, Hongfei Chen, Zhijie Guo, Shuo Zhang, Yihao Wang, Zirui Zhou, Tangwei Li, Lixian Sun

**Affiliations:** 1 Guangxi Key Laboratory of Information Materials, Guilin University of Electronic Technology, Guilin 541004, China; guet_hgz@163.com (G.H.); 19580798291@163.com (L.T.); chfdgryx@yeah.net (H.C.); guozhijie000002@163.com (Z.G.); zhangshuo_0902@163.com (S.Z.); wabgyihao0034@163.com (Y.W.); zhouzirui0522@gmail.com (Z.Z.); litangwei_1222@163.com (T.L.); sunlx@guet.edu.cn (L.S.); 2 Department of Energy, Materials and Telecommunications, INRS-EMT, Quebec, QC J3X 1S2, Canada

**Keywords:** Nb-Ti-Fe alloy, H_2_S poisoning, hydrogen permeability

## Abstract

Currently, the main limitations of Pd-coated Nb-TiFe dual-phase alloys include insufficient hydrogen permeability, susceptibility to hydrogen embrittlement (HE), and poor tolerance of H_2_S poisoning. To address these issues, this study proposes a series of improvements. First, a novel Nb_15_Ti_55_Fe_30_ alloy composed of a well-aligned Nb-TiFe eutectic was successfully prepared using directional solidification (DS) technology. After deposition with a Pd catalytic layer, this alloy exhibits high hydrogen permeability of 3.71 × 10^−8^ mol H_2_ m^−1^ s^−1^ Pa^−1/2^ at 673 K, which is 1.4 times greater than that of the as-cast counterpart. Second, to improve the H_2_S corrosion resistance, a new Pd_88_Au_12_ catalytic layer was deposited on the surface using a multi-target magnetic control sputtering system. Upon testing in a 100 ppm H_2_/H_2_S mixture, this membrane exhibited better resistance to bulk sulfidation and a higher permeance recovery (ca. 58%) compared to pure Pd-coated membrane. This improvement is primarily due to the lower adsorption energies of the former with H_2_S, which hinders the formation of bulk Pd_4_S. Finally, the composition region of the Pd-Au catalytic membrane with high comprehensive performance was determined for the first time, revealing that optimal performance occurs at around 12–18 at.% Au. This finding explains how this composition maintains a balance between high H_2_ permeability and excellent sulfur resistance. The significance of this study lies in its practical solutions for simultaneously improving hydrogen permeability and resistance to H_2_S poisoning in Nb-based composite membranes.

## 1. Introduction

Producing H_2_ from fossil fuels has become one of the most cost-effective methods for large-scale production of hydrogen today [1,2]. In this context, hydrogen production technologies, such as the electrolysis of water and coal gasification, have emerged as essential solutions [3,4]. However, a significant drawback of these approaches is the presence of impurity gases (e.g., CO, CO_2_, CH_4_, and H_2_S) in the syngas, which must be further purified to obtain high-purity hydrogen [5]. To meet this need, Pd-alloys have attracted increased attention and have been applied in these processes due to their unique selectivity to H_2_ via a solution-diffusion mechanism [6,7]. Unfortunately, Pd resources are limited and very expensive, which limits its widespread application [8]. This highlights the urgent need to explore alternatives such as low-Pd (e.g., ever-decreasing thickness [9]) or non-Pd alloy membranes.

**Figure 1:**
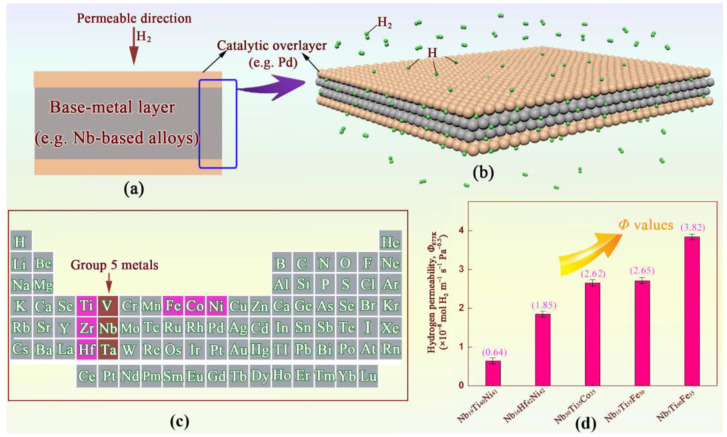
Design concept and appropriate element selection for group 5B composite membranes: (**a**) the unique sandwich-like Pd/base-metal/Pd structures; (**b**) schematic of hydrogen permeation in the sandwich-like membranes; (**c**) the positions of the constituent elements of hydrogen permeable alloys in the periodic table of elements; and (**d**) comparison of hydrogen permeability of several representative Nb-based alloy membranes.

In this context, group 5B alloys (Nb-, V-, and Ta-based [10,11]) have been extensively studied due to their two-fold beneficial characteristics of low price and high hydrogen permeability. Similar to Pd, they also have unique selectivity for hydrogen via a solution-diffusion mechanism, as shown in Figure 1a,b. Until now, two main research directions have been of particular interest: (i) compositional modification/design; and (ii) the development of new process techniques. In terms of compositional design, several classes of alloy have been developed, including amorphous alloys, bcc alloys (body-centred cubic: there is an atom at each corner of each crystal cell and an atom at the centre of each cell), multi-phase alloys, and more recently, high-entropy alloys [12,13,14]. For new processes, technologies such as directional solidification, rolling, and melt-spinning have been developed and applied for bench-scale hydrogen separation [15,16]. Other methods of modifying structural features have also been reported recently. Starting from classical doping of membrane alloys [17] and ending with more modern methods of surface modification such as oxidation in the air [18], modification of the surface of palladium hydrogen-permeable membranes with nanoparticles [19], vacuum deposition [20], and electroless plating [21] chemical vapour deposition [22], etc. To achieve performance improvement, Pushankina et al. [23] recently reported the combined use of the two or three methods mentioned above, as this provides greater flexibility in alloy synthesis and the possibility of creating nanostructured thin films. Regardless of the approach, all of these research efforts align with the U.S. Department of Energy (DOE) goals, aiming to meet the performance targets of temperature (623–773 K), H_2_ flux (not less than 150 cm^3^ cm^2^ min^−1^), overall production cost (below USD 1000 per m^2^), and stability (above 5 years).

Although group 5B alloys have advantages in terms of meeting cost targets, their development is still hindered by challenges related to durability and hydrogen embrittlement resistance (HE resistance). To address these issues, one or more transition metals (such as Fe, Ti, Ni, Co, etc.) were doped internally to alleviate the above problems (Figure 1c). Correspondingly, some binary, ternary, or quaternary systems (e.g., Nb-W/Mo [24,25], Nb-Ti-Fe [26], Nb-Ni-Zr [27]) have recently been developed by researchers. For example, Yukawa, Awakura, Ishikawa, and Dolan.

Over the past decade, our research team has investigated the manufacturing and hydrogen permeability of some dual-phase bcc-cP2 (CsCl prototype) alloys, including Nb-Hf-Co [28], Nb-Ti-Fe [29], and Nb-Ti-Co [30], among others. In particular, Nb-Ti-Fe alloys, primarily composed of inexpensive elemental Fe, are more attractive in terms of cost compared to other Nb- and Pd-based alloys (Figure 1d). Hydrogen permeability is realised with the bcc-Nb solid-solution phase, while the eutectic structure, especially TiFe, suppresses the HE. Consequently, these alloys, such as Nb_5_Ti_60_Fe_35_, Nb_10_Ti_55_Fe_35_, and Nb_15_Ti_55_Fe_30_, exhibit better HE resistance and reasonable H_2_ permeability of (3.5 to 4.1) × 10^−8^ mol H_2_ m^−1^ s^−1^ Pa^−1/2^. Nevertheless, compared to the U.S. DOE’s flux criteria, significant improvements in hydrogen permeation properties are still needed.

As mentioned earlier, compositional modifications appear to be a feasible approach, but they are limited by factors such as the small TiFe phase region and narrow bcc-(Nb, Ti)/TiFe eutectic valley, as demonstrated by our recently calculated Nb-Ti-Fe phase diagram. An alternative solution is to apply advanced fabrication techniques to obtain a hydrogen-permeable alloy with a new structure. To this end, we have tried various methods to prepare novel Nb-Ti-Fe alloy materials with a suitable structure, one of which is directional solidification technology. If the eutectic grains in the membrane are arranged in a regular pattern along the hydrogen permeation direction, ensuring that the Nb phase for hydrogen permeation is uninterrupted, and hydrogen permeation performance (or H_2_ flux) may be significantly improved [31]. However, studies on the directional solidification of Nb-Ti-Fe alloys have not been reported and the effect of this structure on hydrogen permeability is not known.

In addition, Pd-coated Nb-based membranes will quickly (within seconds) lose most of their hydrogen permeation properties to syngas containing a few ppm H_2_S. Therefore, another important issue is how to improve the susceptibility to sulfur compounds in the presence of H_2_S without reducing or alleviating the deterioration of permeability. Detailed research in this field is still lacking, and much creative work is urgently needed to address these challenges.

With these considerations in mind, the aim of the present study are (1) to investigate the feasibility of directional solidification (DS) technology in improving the hydrogen permeation performance of Nb-Ti-Fe alloy membranes; (2) to elucidate the effect of the co-existing gas (i.e., 1–100 ppm H_2_/H_2_S mixture) on the hydrogen transport performance in terms of permeability and recovery of the H_2_ permeance; and (3) to explore the approach to enhance the sulfur resistance of the Pd-Au catalyst layer and ultimately screen for the appropriate composition range where the membrane shows less permeance loss during poisoning and larger permeance recoverability after H_2_S removal. The importance of this work lies in providing guidance and novel ideas for further developing composite membranes with a sound combination of high hydrogen permeability, excellent HE resistance, and high sulfur resistance.

## 2. Experimental and Numerical Procedures

### 2.1. Sample Preparation

**Figure 2:**
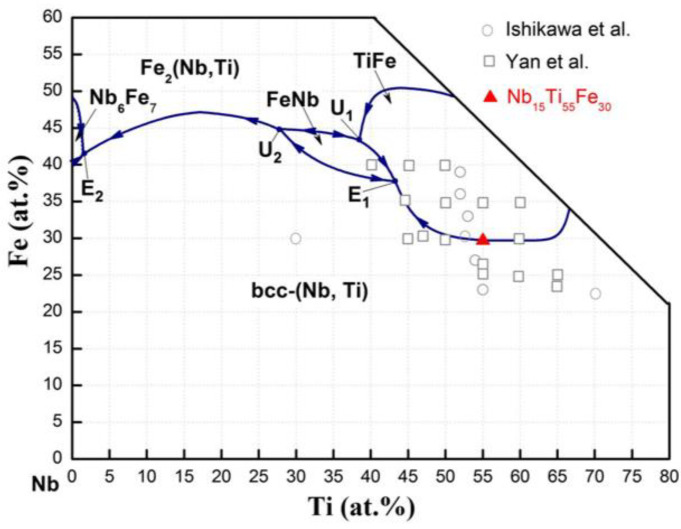
The calculated liquidus projections of Nb-Ti-Fe system and the corresponding position (red solid triangle, ▲) of Nb_15_Ti_55_Fe_30_ alloy in the phase diagram. The previously work of Yan et al. [13] and Ishikawa et al [26] are also shown in the figure.

The Nb (99.99%), Ti (99.99%), and Fe (>99.9%) were weighed according to the alloy composition. All these raw materials were purchased from the Tim New Materials Co., Ltd(Headquartered in Beijing, China). Nb_15_Ti_55_Fe_30_ ingots (ca. 35 g) were fabricated by arc-melting under argon atmosphere. It is worth noting that due to the alloy composition being located in the eutectic groove (see Figure 2), its melting point is relatively low, and its flow ability is good. Therefore, the melting power was controlled at 270 W, which is lower than that of eutectic Nb_30_Hf_35_Co_35_ or Nb_30_Ti_30_Co_30_. To ensure chemical uniformity, each ingot was flipped and re-melted six times. The disk samples (ϕ16 mm × 0.7 mm thick) were cut from the above ingots, which were polished and placed in a drying oven for later use. Additionally, the cylindrical Nb_15_Ti_55_Fe_30_ ingots were fabricated by induction skull melting (ISM) in a purified argon atmosphere in order to conduct subsequent Bridgeman-type DS experiments. Rod-shaped samples (ϕ15 mm × 110 mm length) were extracted from the central region of a cylindrical ingot. One sample was then placed into an alumina tube, with the remaining interstices filled with a high-purity yttrium oxide layer, serving as a protective coating. After vacuuming and leak detection, the sample was heated to 1673 K and stabilised for 30 min. Subsequently, the crucible with the sample was pulled at 1 mm s^−1^ under the temperature gradient of 40 K mm^−1^. After the DS, the crucible was quenched into a liquid metal bath (Ga-25 wt.%In-13 wt.%Sn alloy) at a selected rate. Detailed DS experimental procedures and parameters can be found in our previous work [28]. The longitudinal and transverse sections of the specimens were cut and polished for subsequent characterisation. In addition, the quenched sample was cut into thin slices along the transverse direction for later hydrogen permeation testing. For convenience, it is hereafter referred to as ‘DS-Nb_15_Ti_55_Fe_30_’, as shown in Table 1.

In contrast, the samples prepared by arc-melting, as mentioned earlier, were abbreviated in the form of ‘AC-Nb_15_Ti_55_Fe_30_’ in this work. Both surfaces of each sample were deposited with a thin Pd or PdAu coating (ca. 200 nm) by DC-magnetron sputtering to prevent oxidation during the hydrogen permeation test and to investigate the resistance to H_2_S poisoning. Before depositing the film, each membrane was washed with acetone, dried at 150 K for about 30 min, and then was sent into the sputtering chamber. A 5″ × 12″ Pd sputter target with Au foil tiles placed on the surface of the target was utilised to control Pd-Au composition. Throughout the entire sputtering process, the working pressure for sputtering was maintained at around 1.6 Pa, and the substrate temperature was controlled at 573 K. The distance between the substrate holder and the target is about 60 mm. To obtain the ideal coating and ensure uniformity, the substrate is rotated at a uniform speed and the two targets Pd and Au can be controlled separately during the sputtering process. After the coating sputtering process is completed, each membrane was annealed at 623 K in argon for 2 h to relieve residual stress.

### 2.2. Hydrogen Permeation Test

The hydrogen permeation performance of the as-prepared membranes was measured using a constant-pressure gas permeation technique. The membranes were compression-sealed between two annealed copper washers, with the permeation module having an inner diameter of 12 mm, an outer diameter of 16 mm, and an effective permeation area of 1.13 cm^2^. After vacuum leak detection, pure H_2_ gas permeation was carried out at the set temperature. After measuring for 3 h, the membranes were exposed to an H_2_/H_2_S mixture at 673 K. The range of variation in H_2_S concentration varied from 1 ppm to 100 ppm. During the hydrogen permeation test, the permeate side was fixed at atmospheric pressure. The H_2_S inhibition tests lasted for approximately 8 h, followed by hydrogen recovery in pure H_2_. Finally, pure Ar was introduced on the feed side to check the integrity of the composite membrane after each set of experiments. The H_2_ flux (*J*) was measured by a flow meter. The hydrogen permeability can be obtained using the following equation:

(1) $$J=\frac{\Phi (P_{u}^{0.5}-P_{d}^{0.5})}{L}=\frac{D\cdot K(P_{u}^{0.5}-P_{d}^{0.5})}{L}$$

where *L* represents the final thickness of the sample, *P* represents H_2_ pressure, and subscripts ‘u’ and ‘d’ correspond to feed and permeate sides, respectively. *Φ* is the hydrogen permeability and is equal to the product of hydrogen diffusion rate (*D*) and solubility (*K*).

### 2.3. Sample Characterisation

The composition of the Pd or Pd-Au catalytic film was measured using electron probe microanalysis (EPMA) to ensure they met design requirements. The surface morphology and microstructural characteristics of the matrix alloy were analysed by scanning electron microscopy (SEM, FEG Quanta-450, Hillsboro, Oregon, USA) equipped with an energy-dispersive X-ray spectrometer (EDS). Correspondingly, the types of constituent phases were analysed by X-ray diffraction (XRD, D8 Advance, Bruker, Karlsruhe, Germany) with the scanning 2-theta range of 20–90° (step size: 0.02°). The crystal orientation of constituted phases for as-cast or DS samples was also analysed by transmission electron microscopy (TEM, Talos-F 200X, FEI, Tokyo, Japan).

### 2.4. Computational Methods

The geometry of H_2_ or H_2_S adsorption on Pd and PdAu was constructed by using first-principles density functional theory (DFT) total energy calculations [32], performed with the Vienna Ab initio Simulation Package (VASP) code. During the model construction process, a three-dimensional (3D) infinite periodic structure was used to approximate the crystal cell structures of Pd and PdAu (4 × 4 × 4 k-grid sampling). When simulating the adsorption of S on a (100) surface, S atoms were placed on the same side of the atomic layer, and the relevant parameters can be found in Ref [33]. Correspondingly, the adsorption energy of gas molecules adsorbed on (100) surfaces was calculated. The diffusion activation energy and minimum diffusion path of H atoms in the octahedral interstitial site (OIS) and the tetrahedral interstitial site (TIS) were calculated by using the climbing image-driven nudge elastic band method coupled with the Arrhenius diffusion equation. In the above calculation process, VASP code and generalised gradient approximation. Prior to simulation, model validation was conducted, and the constructed model demonstrated the capacity to reflect the behavioural characteristics of the real system. The Perdew–Burke–Ernzerh (PEB) method was also utilised to describe the exchange correlation between electrons.

## 3. Results and Discussion

**Figure 3:**
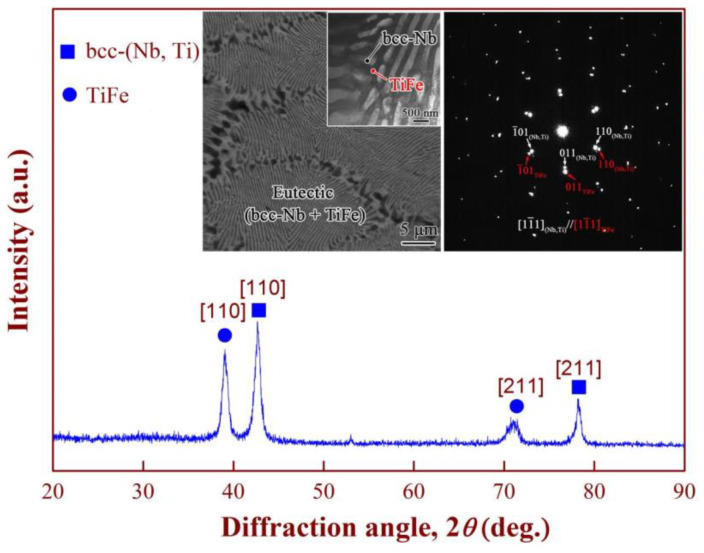
XRD patterns of the as-cast Nb_15_Ti_50_Fe_35_ alloy. Insets show the multi-phase microstructure (**left**) and the selected area electron diffraction (SAED) patterns (**right**) taken from the bcc-Nb and TiFe phases, respectively.

Figure 3 shows the structural characteristics of the as-cast Nb_15_Ti_55_Fe_30_ alloy. Two diffraction peaks of TiFe and bcc-Nb phases are identified by XRD analysis, consistent with the SEM micrograph; see the left inset in Figure 3. The intensity of the [110] Bragg peaks is relatively high at 39.01° and 42.63°, while the measured [211] diffraction peaks at 71.05° and 78.23° are relatively weak. This indicates that the relevant phases or grains grow along the preferred orientation in the <211> direction. The orientation relationship (OR) between the TiFe phase and bcc-Nb phase was further determined by the selected area electron diffraction (SAED) patterns (the right inset in Figure 3). These two phases have the same crystallographic (cubic) structure but belong to different space groups, namely Pm3m for the TiFe and Im3m for the bcc-Nb phases. The results show that the TiFe and bcc-Nb phases have consistent cubic-on-cubic OR configuration: [110]_bcc-Nb_/[110]_TiFe_ or [001]_bcc-Nb_/[001]_TiFe_. This orientation may have been produced by eutectic coupling growth during non-equilibrium solidification (bcc-Nb + TiFe). The formation of a fully eutectic structure is attributed to the alloy composition being located at the eutectic valley position of L → bcc-Nb + TiFe, plus the binary eutectic solidification termination being before the ternary eutectic point E_1_.

Moreover, compared to eutectic Nb_30_Ti_35_Co_35_ and Nb_19_Ti_40_Ni_41_, the proportion of bcc- Nb in eutectic Nb_15_Ti_55_Fe_30_ is higher (ca. 34.7 vol.%). As reported by Ishikawa and Awakura et al., the higher the content of the bcc-Nb phase, the greater the number of transport pathways for hydrogen atoms during hydrogen permeation. These results may help explain why eutectic Nb-TiFe exhibit better hydrogen permeability than other Nb-based alloys, as reported in the introduction earlier in Figure 1.

**Figure 4:**
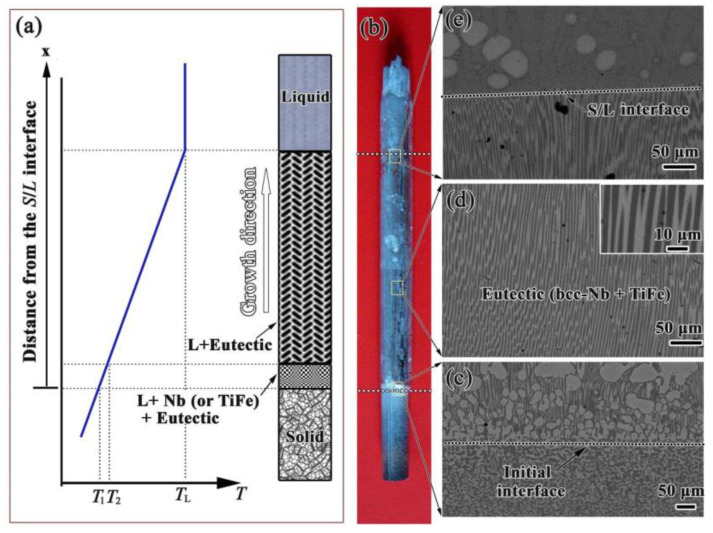
The directional melting process of Nb_15_Ti_50_Fe_35_ alloy and relevant microstructural morphology: (**a**) the schematic of formation of the mushy zone after the imposed temperature gradient inside the rod; (**b**) the DS ingots of Nb_15_Ti_50_Fe_35_ alloy at a growth rate of 1 mm s^−1^; (**c**–**e**) the longitudinal section microstructures corresponding to local regions of initial growth interface, steady-state growth, and quenching interface. Insets in (**d**) show the enlarged longitudinal section microstructures.

Figure 4 shows the overall appearance and the SEM micrographs of the Nb_15_Ti_55_Fe_30_ alloy after the DS experiment solidified at a growth rate of 1 mm s^−1^. When a dual-phase Nb-TiFe alloy rod solidifies in a Bridgman-type furnace, the types of structure in the mushy zone may be subdivided into two categories: hypoeutectic or hypereutectic, and fully eutectic, as seen in Figure 4a. After cutting and polishing the DS Nb_15_Ti_55_Fe_30_ alloy ingot in Figure 4b, the leading bcc- Nb phase at the initial-growth interface is observed (Figure 4c) which implies that the local composition deviates slightly from the eutectic valley during thermal stabilisation, see Table 1.

During directional growth, there is a mush zone between the solid (*S*) zone and the liquid (*L*) zone. This process, known as the preparation of the initial interface, is often observed in the directional solidification process [34]. As directional solidification begins, although a small amount of bcc-Nb crystals precipitate, their growth is soon inhibited by the proximity of the melt to the eutectic composition. As a result, after the initial growth interface, crystals in the steady-state growth zone exhibit a fully eutectic growth, and two well-aligned phases parallel to the growth direction are observed (Figure 4d). These two phases continue growing in this mode until the sample is quenched (Figure 4e).

Interestingly, the microstructure of the directionally solidified sample is entirely composed of rod-shaped eutectic, which is significantly different from the lamellar eutectic in the as-cast counterpart in Figure 3. According to the Jackson–Hunt eutectic growth model, this difference is primarily related to the decrease in the proportion of the TiFe phase in the eutectic for the DS sample.

**Figure 5:**
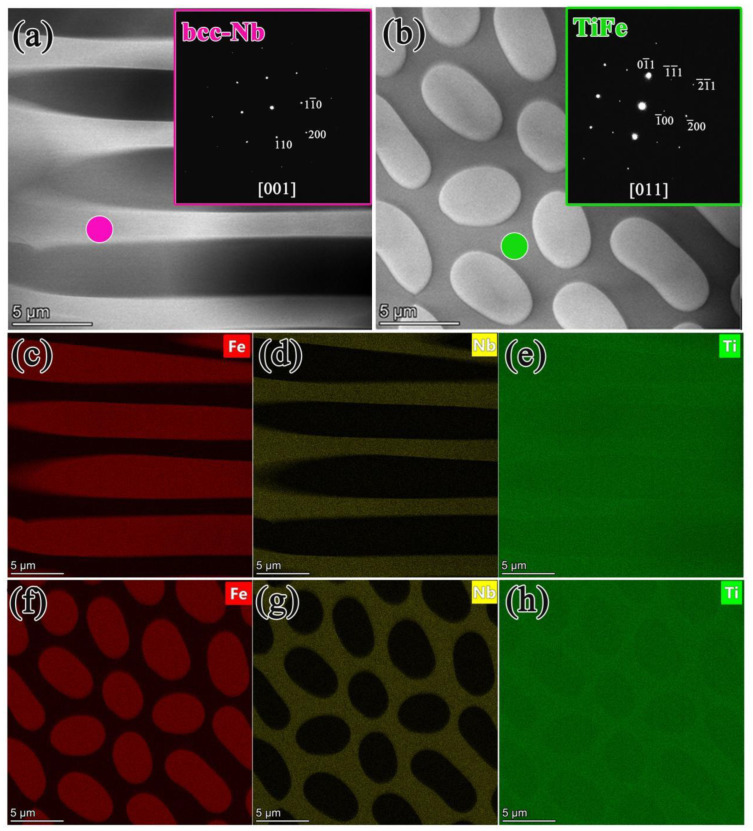
Structural features and orientation relationship between the TiFe phase and Nb phase of directionally solidified Nb_15_T_50_Fe_35_ alloy: (**a**,**b**) the longitudinal and transverse section TEM bright field images of steady-state growth; (**c**–**h**) the distributions of Fe, Nb, and Ti in the longitudinal and transverse cross-sectional structures, respectively. Insets in (**a**,**b**) show the SAED patterns taken from the bcc-Nb and TiFe phases, respectively.

From the TEM bright field images of steady-state growth in Figure 5, the diameters of Nb rods are around 3–5 nm, and they are uniformly distributed in the TiFe matrix. Additionally, no obvious OR can be observed between these two phases. It can be inferred that the directional solidification process changed the morphology and the OR between the TiFe and bcc-Nb phases. The atomic percentage ratio of Nb, Ti, and Fe (X_Nb_:X_Ti_:X_Fe_) in the primary phase is 37.8:46.3:15.9, respectively, and thus the bcc-Nb phase can be identified. The atomic percentage ratios of Nb:Ti:Fe in the TiFe phase are 6.15:44.78:49.07, respectively. The distribution of elements in the longitudinal and transverse cross-sectional microstructure (Figure 5c–h) confirms that the enrichment areas of each element (e.g., Fe, Nb, and Ti) are consistent with the corresponding phase analysis mentioned above.

**Figure 6:**
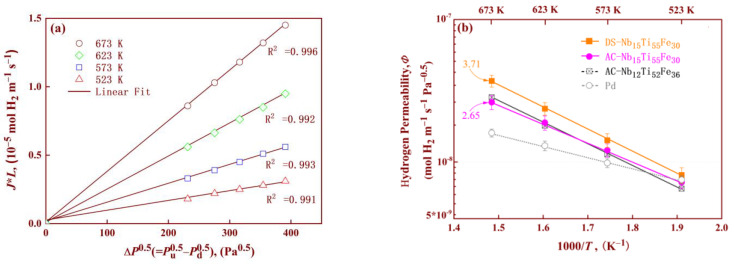
Hydrogen permeabilities of the Nb-Ti-Fe membranes in pure H_2_: (**a**) representative relation between (J*L) and Δ*P*^0.5^ for the DS-Nb_15_T_i50_Fe_35_ alloy; and (**b**) Arrhenius curve between *Φ* and 1/*T* of Pd [9], AC-Nb_12_Ti_52_Fe_36_ [13] and all the Nb-Ti-Fe samples.

Subsequently, permeation measurements were conducted under a pressure difference of 30–50 kPa and a temperature range of 523–673 K for all the samples, and the results are shown in Figure 6. In all cases, the H_2_ flux was observed to yield a linear dependence with pressure difference (Δ*P*^0.5^ = *P*_u_^0.5^ − *P*_d_^0.5^) on both sides of the membrane, indicating that all the membranes studied here conform to the solution-diffusion mechanism. The exponential factor of pressure difference, Δ*P*, is useful for evaluating the rate-limiting step in the hydrogen permeation process. When it is equal to approach 1, the hydrogen adsorption in the membrane surface is the rate-limiting step. In this work, its value is 0.5, which means that hydrogen transport is a diffusion-limited permeation process. Figure 6a shows a typical relation between J*L and Δ*P*^0.5^ for the DS-Nb_15_T_i50_Fe_35_ alloy. Clearly, all the experimental data exhibited good linearity at each temperature after linear fitting, and their correlative coefficients R^2^ are greater than 0.99. Such results further imply that the diffusion process, rather than surface reaction (e.g., hydrogen dissociation or recombination), controls overall hydrogen permeation. In fact, the latter often occurs in ultra-thin (usually at the micrometre level [35]) alloy membranes. When the membrane thickness is reduced to 6 μm, non-diffusive factors such as the hydrogen dissolution reaction on the membrane surface play a certain role in hydrogen permeation, as reported by Zhang [36] in porous Ni-based composite membranes. For the synthesised membranes in this work, their thickness (about 0.7 mm) is much greater than the aforementioned 6 μm, which explains the dominant role of diffusion in the entire hydrogen permeation process. Nevertheless, the critical value of membrane thickness corresponding to the dominant role of diffusion has not yet been reported, mainly because, as the membrane thickness gradually decreases, the probability of breakage increases, leading to unusually cumbersome experiments, as reported by Roa, Nishimura et al. [37,38].

According to Equation (1), the pure H_2_ permeability (*Φ*) for the AC and DS samples at each temperature was obtained by calculating the slopes of the fitted curves in Figure 6a. Correspondingly, the Arrhenius plot of the permeability is shown in Figure 6b. For comparison, the permeability of previously reported Nb_12_Ti_52_Fe_36_ and pure Pd is also depicted in the figure. Clearly, the *Φ* values exhibited a linear dependence with 1/T in the form of an Arrhenius plot, and in all cases, the permeability increased with increasing temperature. This is due to the fact that the passage of H atoms through the membrane is mainly affected by the diffusion rate, which increases with increasing temperature. This can also be concluded from the increase in K in Equation (1). The dependence of permeability on temperature for Nb-Ti-Fe alloys is higher than that of pure Pd, implying that the former has higher activation energies (*E*_a_) for alloy hydrogen permeation. In fact, the values of *E*_a_ for Nb-Ti-Fe alloys were determined to be 24 ± 2 kJ mol^−1^ after linear fitting, which is about double the value (ca. 12.21 kJ mol^−1^) of pure Pd. Furthermore, the permeability of all the Nb-Ti-Fe alloys was superior to that of Pd, especially at temperatures above 573 K. The *Φ* values of DS-Nb_15_Ti_55_Fe_30_ were significantly higher (3.71 × 10^−8^ mol H_2_ m^−1^ s^−1^ Pa^−1/2^ at 673 K) than those of both the as-cast counterpart (Nb_15_Ti_55_Fe_30_) and previously reported Nb_12_Ti_52_Fe_36_ at all test temperatures. After pure hydrogen testing, pure Ar was injected into the permeator, and its flux values were almost undetectable because the flux values were much lower than the detection limit of the flow meter. This indicates that the Nb-Ti-Fe membranes were intact and exhibited infinite hydrogen selectivity. This result also suggests that the membranes were less susceptible to HE, ensuring membrane integrity during hydrogen permeation and preventing crack formation caused by HE.

It has been reported that the factors influencing hydrogen permeability can be assigned to (i) the content of the primary bcc-Nb phase (closely related to alloy composition); (ii) phase morphology and interface; or (iii) crystal orientation between the constituent phases. Generally, high Nb content alloys in the Nb-TiNi/TiCo system crystallise more of the primary Nb phase, which is beneficial for hydrogen permeation. However, this does not apply to the Nb-TiFe system, as alloys that solidify to form primary Nb phases are closer to the Ti-rich corner region. All reported hydrogen permeable Nb-TiFe alloys have relatively high Ti content (ca. 55–60 at.%), while Nb content is relatively low (<10 at.%). He [39] reported that the phase interface can serve as a hydrogen diffusion channel, which is beneficial for increasing hydrogen permeability. On the contrary, Díaz [40] argues that the interface will capture hydrogen atoms, which reduces hydrogen diffusion and deteriorates hydrogen permeability while increasing the risk of HE. These controversies still need further experimental confirmation. Nevertheless, both the fundamental structural models and effective medium theory confirm that the orientation of phase alignment significantly impacts hydrogen permeability [41]. In our case, DS samples with regular phase arrangement have higher permeability, further demonstrating the importance of phase arrangement in controlling performance. In addition, directional solidification also changes the crystallographic orientation between two phases, that is, the ‘cube-on-cube’ OR of the as-cast sample will disappear, which indicates that compared with the phase arrangement, the crystallographic orientation has a smaller impact on permeability, although Ishikawa [42] reports that Nb alloys with the ‘cube-on-cube’ relationship have higher permeability values. In summary, these results demonstrate that directional solidification technology can tailor the microstructure of Nb-TiFe alloy systems, especially the arrangement of phases, thereby improving the hydrogen permeability. Subsequently, membrane permeation tests in H_2_/H_2_S mixtures were conducted, and their performance with respect to the permeability and H_2_S response was gradually measured.

**Figure 7:**
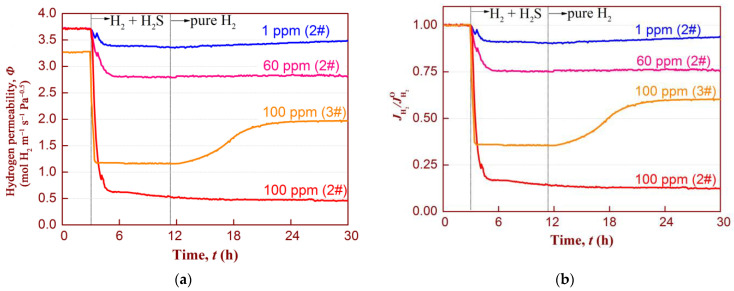
Hydrogen permeation performance of the Pd-(2#) and Pd_88_Au_12_-coated (3#) membranes after introduction of 1 ppm, 60 ppm, and 100 ppm H_2_S at 673 K for 8 h and the subsequent recovery in pure hydrogen: (**a**) permeability; and (**b**) relative H_2_ flux (*J*/*J*^O^).

Figure 7 shows the flux inhibition upon exposure to H_2_S and subsequent recovery in pure H_2_ for all the Nb-Ti-Fe alloy membranes. Note that samples 2# and 3# were coated with pure Pd and Pd_88_Au_12_ films on their surfaces, respectively. According to the standards of the U.S. DOE, the H_2_S concentration variation range is selected from 1 ppm to 100 ppm (Figure 7a). The relative H_2_ permeability (*J*_H2_/*J*^O^_H2_) shown in Figure 7b is defined as the ratio of H_2_ flux under mixed atmosphere (*J*_H2_) and pure hydrogen gas (*J*^O^_H2_). The Pd-containing membranes (2#) were first measured in ascending order of H_2_S concentration at 673 K. After feeding 1 ppm H_2_S to the reactor, a sharp drop in permeability was observed, followed by a slow decrease, ultimately reaching a stable state after 6 h at a value of approximately 3.38 × 10^−8^ mol H_2_ m^−1^ s^−1^ Pa^−1/2^. As the H_2_S concentration increased, the permeability continued to decline, as shown in Table 2. The permeability was only 0.5 × 10^−8^ mol H_2_ m^−1^ s^−1^ Pa^−1/2^ at 100 ppm H_2_S, and the drop ratios in H_2_ permeance were approximately 87% (Figure 7b). This indicates that the degradation of permeability is closely related to the H_2_S concentration, and the higher the concentration, the greater the permeance degradation. Furthermore, the dual reduction in the permeability upon H_2_S exposure was more than just competitive adsorption of sulfur and hydrogen atoms on the Pd site. In addition to the initial sharp decline in permeability caused by surface site blocking of dissociation sites of H_2_ molecules accompanied by sulfur adsorption, the subsequent slow decline in permeability may be closely related to the formation of bulk palladium sulfides (mostly Pd_4_S) with exposure time. Since the permeability of Pd_4_S is two orders of magnitude lower than that of Pd [43] at temperatures between 600 K to 1200 K, the permeability gradually decreases as the thickness of the Pd_4_S reaction layer increases over time.

After 8 h of H_2_S exposure at 673 K, all the Pd-containing membranes were fed again with pure H_2_. At all tested concentrations, the lack of obvious changes in permeability for up to 18 h suggests almost no permeance recovery, likely due to the formation of irreversible bulk sulfides. In contrast, McKinley et al. [44] stated that the Pd foil showed a hydrogen recovery of almost 100% after exposure to H_2_/H_2_S (4.5 ppm) mixture at 623 K, with no signs of sulfide formation observed on the membrane surface under their testing conditions. Some important experimental parameter differences, for example, H_2_S concentration or testing temperature, may explain the differing results. Another reason could be the different critical thermodynamic conditions for the formation of irreversible palladium sulfide on the membrane surface at different temperatures. Mundschau et al. [45] used thermodynamic calculations to show that some irreversible bulk Pd_4_S is formed within Pd when H_2_S concentration exceeds the limit concentration of 1 ppm at 623 K. These calculated results seem to contradict those of McKinley et al. but are consistent with our findings.

Figure 7 also shows that the evolution of permeability under exposure to 100 ppm H_2_S and subsequent recovery in pure H_2_ for the Pd_88_Au_12_-coated membrane (3#). Preliminary observations suggest that this membrane also experienced a deterioration in hydrogen permeability, similar to the Pd-coated membranes mentioned above. Nevertheless, this membrane exhibited a higher permeability value in the presence of H_2_S than the Pd-coated membranes. Also, the permeance recovery is fast and high (ca. 58%) after being fed pure H_2_ again. This suggests that the performance degradation is mainly caused by the membrane surface site blocking due to H_2_S desorption, rather than the formation of bulk sulfidation, as the recovery of permeability is often related to the desorption of adsorbed sulfur. The introduction of hydrogen will gradually reduce the concentration of H_2_S, thereby affecting the gas–solid equilibrium of H_2_S dissociation and adsorption, ultimately leading to sulfur desorption and the recovery of permeability.

However, the permeance recovery rate (slope) followed a pattern of rapid initial increase (ca. 40% of the original value), which then slowed down and eventually remained constant. This is likely related to the fact that when adsorbed sulfur enters the Pd-Au site, the binding energy changes. As reported by Mejdell et al. [46], in their theoretical calculations, the reduction in sulfur coverage on the Pd surface increases the binding energy of sulfur/Pd and reduces the repulsive force between sulfur atoms, making it more difficult for sulfur to escape the Pd surface. Therefore, in the later stages of recovery, as the sulfur coverage decreases, the permeance recovery rate shows a lower value.

**Figure 8:**
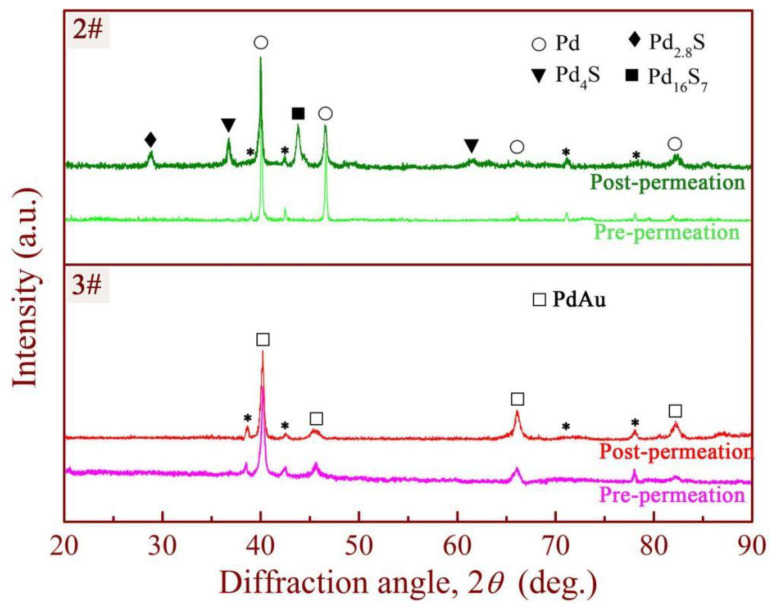
The X-ray diffraction patterns of Pd-(2#) and Pd_88_Au_12_-coated (3#) membranes after exposure to 100 ppm H_2_S (* represents matrix phases).

X-ray diffraction patterns (Figure 8) indicate no formation of a bulk sulfide phase on the Pd_88_Au_12_-coated membrane (3#). In contrast, three sulfides (Pd_4_S, Pd_2.8_S, and Pd_16_S_7_) were detected by XRD for Pd-coated composite membrane 2#. This indicates that the Pd_88_Au_12_-containing film displays enhanced chemical stability and exhibits greater resistance to sulfur toxicity in comparison to the Pd-coated film.

**Figure 9:**
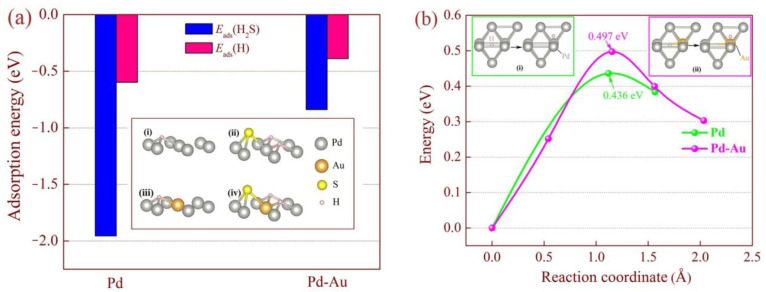
The absorption and diffusion of hydrogen molecules and hydrogen sulfide molecules in Pd and PdAu alloys (DFT results): (**a**) absorption energy; and (**b**) diffusion barrier energy of an H atom diffusing across TIS and the nearest OIS.

To reveal the underlying mechanism of anti-H_2_S poisoning in the Pd_88_Au_12_-coated membrane, density functional theory was employed to investigate the adsorption energy and diffusion activation energy of H_2_S and H_2_ on Pd or PdAu surfaces, as shown in Figure 9. When polar H_2_S molecules and non-polar H_2_ molecules adsorb on the clean Pd(001)-s surface, the most stable positions correspond to the hollow site (HS) and the top site (TS), respectively. Both exhibit similar stable adsorption sites when adsorbed on the PdAu(001)-s surface. Nonetheless, Au substitutions in Pd increase the adsorption energies (*E*_ads_) of H_2_ (−0.58 eV → −0.47 eV) and H_2_S (−0.196 eV → −0.92 eV), with the latter being more pronounced (Figure 9a). This indicates that pure Pd has a stronger affinity for H_2_S molecules in comparison to PdAu alloys, resulting in easier adsorption on Pd alloys due to its lower *E*_ads_.

When H_2_S is fed to the membrane, it quickly occupies the surface site, thereby reducing the effective active area for hydrogen permeation. In this case, the solubility of hydrogen decreases dramatically, leading to a decrease in permeability, and the subsequent formation of Pd sulfide, further deteriorating permeability. However, doping Au in Pd can mitigate this undesirable condition, as the adsorption energy of H_2_S on PdAu sites makes it more difficult for the molecules to adsorb on the surface. This may help to explain why PdAu-containing membranes exhibit higher resistance to H_2_S corrosion under H_2_S-containing mixed gas, as mentioned earlier in Figure 7. Furthermore, Morreale et al. [47,48] reported that the enthalpy of the formation of PdS is lower than that of AuS at 673 K, suggesting that the replacement of Pd with Au in alloys would improve the resistance to sulfide formation.

Moreover, it is widely acknowledged that the migration of hydrogen in metals is typically accomplished through a sequence of transitions between tetrahedral interstitial sites (TIS) and octahedral interstitial sites (OIS) [49]. Some defect locations (such as dislocations and grain boundaries [50]) are also reported to have similar functions. Among all diffusion paths in pure Pd, the diffusion energy barrier from the TIS to the nearest OIS is the lowest (0.436 eV, see Figure 9b); thus, TIS → OIS is the preferred path, which is in good agreement with the calculated results of Xu and Zhao et al. [51,52].

However, the substitution of Au in Pd significantly increases the diffusion energy barrier (0.436 eV → 0.497 eV), although it has little effect on the path length of hydrogen diffusion. At the TIS of the PdAu lattice, active hydrogen atoms are in a metastable state, and their diffusion to the adjacent OIS requires overcoming a larger energy barrier. In other words, hydrogen diffusion in the PdAu lattice is more difficult compared to pure Pd, and doping Au in Pd is unfavourable for hydrogen permeation to some extent. This may be a contributing factor in the reduction in pure H_2_ permeability of the PdAu-coated membrane, as illustrated in Figure 7a.

**Figure 10:**
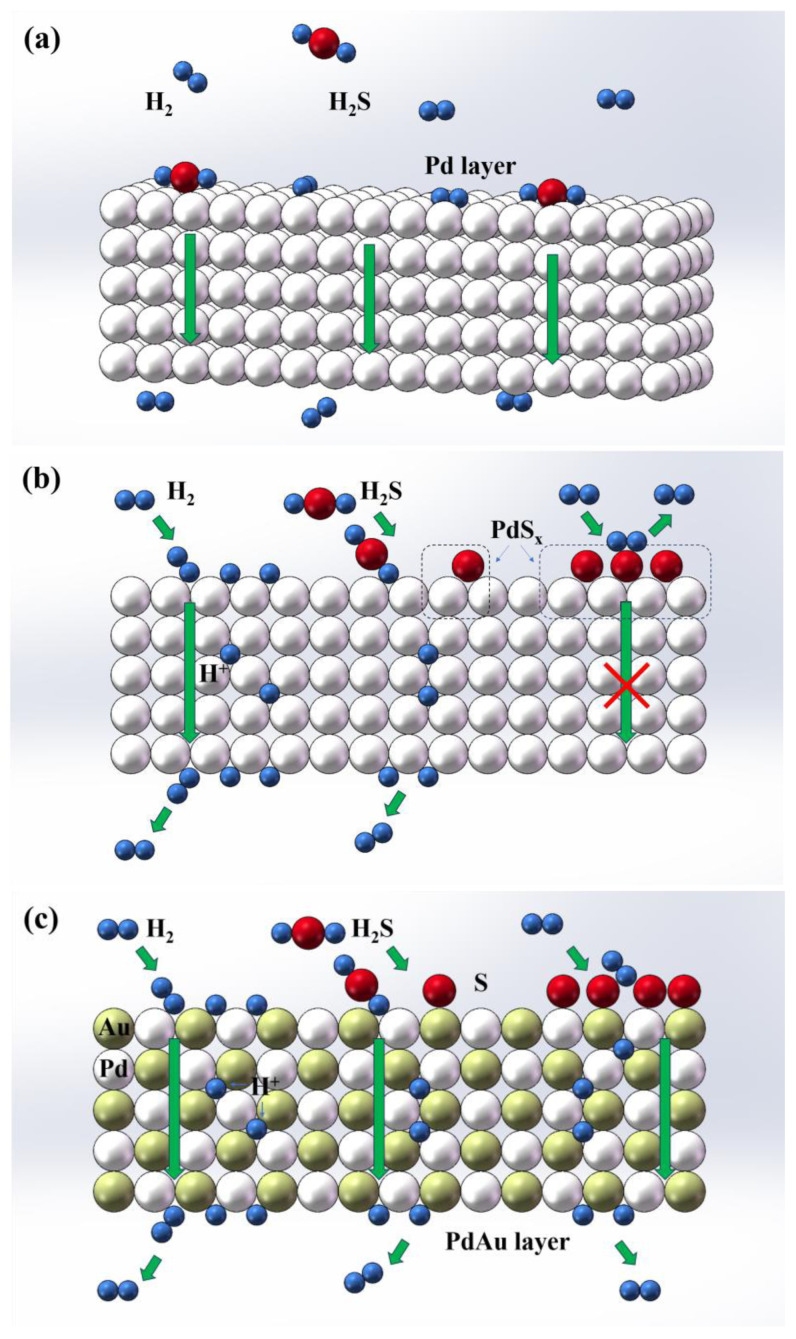
A schematic drawing of hydrogen permeation in the outermost catalytic layer: (**a**) the dissociative adsorption/combination and desorption of H_2_ or H_2_S molecule; (**b**,**c**) represent atomic H diffusion in pure Pd and PdAu alloy films, respectively.

In short, these calculated results generally align with the experimental values, collectively substantiating that doping Au in Pd can significantly improve the tolerance of the composite membrane to poisoning with H_2_S. The schematic diagram in Figure 10 provides a clear explanation of this phenomenon.

After H_2_S molecules are adsorbed onto the Pd surface (Figure 10a), they gradually dissociate into sulfur atoms and hydrogen molecules according to the following four steps [53]:

(i) H_2_S (g) + * ↔ *SH_2_ (ad)

(ii) *SH_2_ (ad) + * ↔ *SH (ad) + *H (ad)

(iii) *SH (ad) + * ↔ *S (ad) + *H (ad)

(iv) *H (ad) + *H (ad) ↔ H_2_ (g) + 2*

where the symbols ‘g’, ‘∗’, and ‘ad’ represent the gas, surface site, and a different adsorbed intermediate, respectively. The overall reaction for the above four steps is as follows:

(v) H_2_S (g) + * = *S(ad) + H_2_

On the one hand, these metastable sulfur atoms quickly occupy the activation position (each adsorbed sulfur atom blocks approximately 4–13 H_2_ adsorption sites [54]) and block surface sites for hydrogen permeation. On the other hand, they react with Pd atoms to form sulfides after meeting specific thermodynamic conditions; see the circle position in Figure 10b. With the changes in thermodynamic and kinetic conditions, although the former is reversible and dynamically releases active sites, the latter process is irreversible, and the generated irreversible bulk sulfidation, as a mass transfer barrier, causes the membrane to permanently lose its hydrogen permeability. In addition, the membrane is often at risk of rupture due to the large difference in lattice constants between the two, which generates significant stresses. In contrast, doping Pd with Au can significantly increase the adsorption energies of H_2_S (Figure 9a), thereby inhibiting the adsorption and dissociation of H_2_S on the PdAu surface. As a result, there are insufficient sulfur atoms available for surface reaction sites to form, as illustrated in Figure 10c. In this case, only a small amount of S atoms reversibly blocks the active sites and cause a decrease in permeability, which disappears with the reintroduction of pure hydrogen. Other doping elements such as Cu also have similar characteristics and offer some sulfur resistance [55]. However, as phase transition from the fcc (face-centred cubic: each cell has an atom at each corner and each face has an atom at its centre) to the bcc structure in Cu-Pd alloys during high-temperature hydrogen permeation (especially temperatures above 550 °C), these catalytic membranes still have significant room for improvement in large-scale methane steam reforming for hydrogen production at high temperatures around 800 °C.

In contrast to PdCu alloys, PdAu alloys are capable of complete miscibility in both liquid and solid states. This suggests that a second phase cannot precipitate in the infinitely miscible PdAu alloy, even at high temperatures. Consequently, the alloy is more stable and suitable for use in high-temperature hydrogen permeation processes, irrespective of the presence or absence of impurity gases.

**Figure 11:**
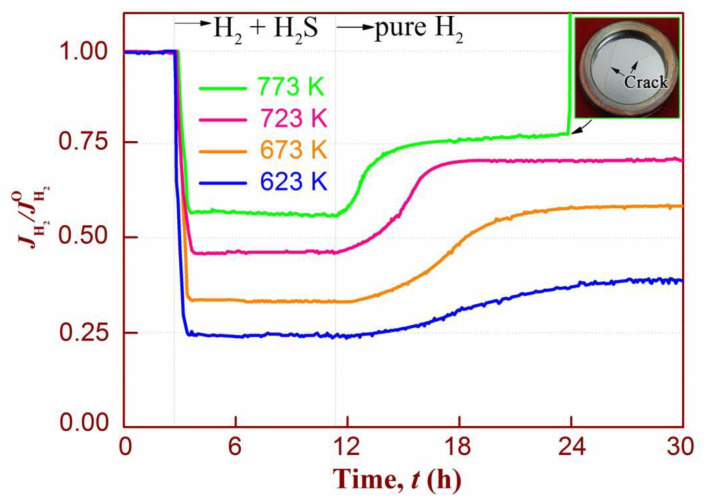
The poisoning and recovery of PdAu-coated (3#) membranes after exposure to 100 ppm H_2_S under temperatures from 623 K to 773 K.

To verify this, high-temperature tests (723 K and 773 K) of membrane 3# in H_2_/H_2_S gas mixture and subsequent recovery in H_2_ were conducted, as shown in Figure 11. For comparison, low-temperature testing at 623 K is also included in the figure. At all testing temperatures, the permeability after exposure to H_2_S experiences an instantaneous decrease and reaches a steady-state value. The higher the temperature, the smaller the decrease in hydrogen permeability. This can be attributed to the dissociation adsorption of H_2_S on the membrane surface, which produces a surface site-blocking effect (see Figure 9a). The thermal motion of H_2_S molecules at high temperatures becomes stronger [56], leading to a decrease in their ability to adsorb on the PdAu surface. In this case, compared to H_2_, H_2_S impurities will gradually lose their adsorption advantage.

Some cracks, possibly caused by HE, were observed on the surface of the membrane after testing at 773 K for 24 h, which provides a reasonable explanation for the instantaneous increase in the relative H_2_ flux of this membrane. Additionally, the hydrogen permeation recovery phenomenon (similar to the result at 673 K) was also observed for all three cases after the membrane was fed pure H_2_. Higher temperatures are more favourable for permeance recovery, which is closely related to the exothermic nature of H_2_S dissociative chemisorption onto PdAu metal. In addition, the gas–solid adsorption equilibrium of sulfur shifts towards the gas phase at higher temperatures, making the dissociation of adsorbed sulfur more favourable, resulting in higher permeability recovery.

Notably, the membranes containing the Pd_88_Au_12_ catalytic layer show a higher permeance loss and lower permeance recovery when compared to the self-supported Pd_68_Cu_32_ alloy foil [57]. All data are tabulated in Table 3. The percent inhibition of permeability is only 10%, and a high permeance recovery of 88% was observed for Pd_85_Au_15_ alloy foil reported in the previous work of Huang et al. [58]. Lewis et al. [59] reported that the Pd_77_Au_23_ alloy recovered hydrogen almost completely when H_2_S was removed from the permeator.

In contrast to the aforementioned PdAu membrane, the Pd_80_Au_20_ [65] membrane shows higher permeability loss and flux inhibition in the presence of H_2_S, and the permeance recovery did not change significantly after removing H_2_S. Some similar cases can be found in other Pd/Au membranes with lower Au content (usually less than 15 at.%). The most likely reasons for this observation are the different feed gas (e.g., H_2_S concentration), H_2_S exposure time, and fabrication technique (ELP, MS, or CW) throughout the entire experimental cycle. Although a direct comparative analysis of hydrogen permeability is difficult due to differences in experimental parameters (H_2_S concentration or exposure time) in the literature, membranes containing high Au content appear to have better resistance to H_2_S in H_2_/H_2_S mixed atmospheres. To confirm this, more tests need to be conducted on PdAu alloy films with a wider range of Au content under similar conditions. Considering this, we made our first attempt to evaluate the comprehensive performance of these composite membranes in the presence of H_2_S by only changing the PdAu composition, yielding good results.

**Figure 12:**
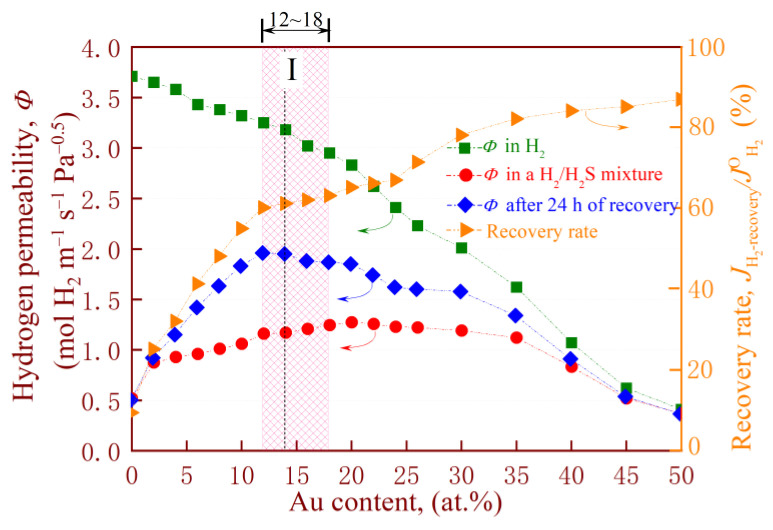
Permeability during the pure H_2_ and the H_2_/100 ppm H_2_S at 673 K feeds and its values after 24 h of hydrogen recovery, as well as the recovery rate of permeability as a function of catalytic layer composition.

Figure 12 shows the data obtained from a composite membrane with 28 components of the PdAu binary alloy catalytic layer. For convenience, these alloys are hereafter referred to as the Pd_88−x_Au_12+x_ (x = −10, −8, …, 38) series, and their permeability (*Φ*) in pure H_2_ and an H_2_/100 ppm H_2_S mixture are represented by solid boxes (■) and solid circles (●), respectively. Correspondingly, the *Φ* values after 24 h of hydrogen recovery (♦) and the recovery rate of *Φ* (►) are also depicted in the figure. Here, the recovery rate of permeability (*J*_H2_-recovery/*J*^O^_H2_) is defined as the ratio of H_2_ flux after 12 h of hydrogen recovery (*J*_H2_-recovery) and pure hydrogen gas (*J*^O^_H2_), which represents the ability of the membrane to recover to its initial pure H_2_ permeability.

Furthermore, experimental parameters such as H_2_S concentration and exposure time are consistent with the membrane testing conditions in Figure 7 above. Clearly, pure H_2_ permeability decreases with increasing Au content, but the recovery rate gradually increases in a pure hydrogen atmosphere after removing H_2_S. This is mainly due to the fact that low Pd content is not beneficial for obtaining high hydrogen solubility, thereby providing a lower driving force for membrane hydrogen permeation. On the other hand, Au provides the major contribution to the prevention of S poisoning, as the binding energy between S and Au is lower than that of Pd, making it less likely to generate sulfides. However, this does not mean that the higher the Au content in the PdAu film, the better its overall performance. When the Au content exceeds 14 at.% (especially 18 at.%), the sharp decrement in pure H_2_ permeability leads to a decrease in the permeability of the membrane in a mixed H_2_/H_2_S atmosphere. After removing H_2_S, the recovered permeability also faces a similar situation.

Additionally, low Au content (e.g., below 12 at.%) can also lead to a decrease in permeability in the presence or absence of H_2_S due to insufficient sulfur resistance of the membrane. In the H_2_/H_2_S mixture, the gold content in the catalytic film was in the range of 12–18 at.% Au; see region I in Figure 12. When Au content is less than 12 at.%, although the membrane has good pure H_2_ permeability, its resistance to H_2_S poisoning is weak, resulting in a low corresponding recovery rate. In contrast, when the Au content exceeds region I, especially Au > 25 at.%, the pure H_2_ permeability sharply decreases, resulting in a decrease in the recovery rate of the alloy. Thus, it is inferred that region I (Au content between 12–18 at.%) is the best choice when selecting a PdAu alloy as the catalytic layer for a hydrogen separation membrane, as it allows for a combination of high permeability and good resistance to H_2_S.

The identification of region I reveals why the Au content in a Pd-Ag alloy, which serves as a catalytic membrane [68], needs to be maintained at a medium-low level, i.e., 12–18 at.%. Alloys with too low and too high Au content will face the risk of insufficient sulfur resistance and deterioration of hydrogen permeation performance in H_2_/H_2_S mixtures. For example, Chen [64] reported the sharp decrease in hydrogen permeation performance of an ultra-thin freestanding PdAu membrane with Au content below 20 at.% upon exposure to 54.8 ppm H_2_/H_2_S mixed gas. This phenomenon may be closely related to the solid solution of Au content in the Pd lattice. A similar loss of permeability after H_2_S exposure was observed in Pd_40_Cu_60_ [57] and Pd_77_Ag_23_ [61,62] alloys. The content and type of solid solution elements in Pd alloys can affect hydrogen dissolution and diffusion, thereby affecting permeability, as confirmed by Kusada [69]. For example, Pd-Cu alloys with a bcc structure are beneficial for hydrogen permeation, while those with the fcc structure exhibit durability against sulfur poisoning. The phase transition from fcc to bcc structure alters their comprehensive properties. Nevertheless, due to the homogeneous phase diagram of the PdAu alloy series, the change in properties due to this phase transition is not applicable to the palladium series. Thus, this implies that the effect of solid solution on performance may be more pronounced for PdAu alloys.

Regardless, these cases further demonstrate the rationality and availability of the newly explored region I in predicting alloy properties upon exposure to an H_2_/H_2_S mixture. Additionally, as recently reported by Peters [62] et al., some Pd-based ternary and quaternary alloy membranes (e.g., Pd_75_Ag_16_Au_9_ [60], Pd_76_Ag_22_Mo_3_ [61], Pd_77_Ag_10_Cu_13_ [70], etc.) show a higher hydrogen recovery within shorter recovery times after H_2_S removal. This implies that doping the third or fourth component into the catalytic membrane is beneficial for improving hydrogen permeability, though this needs more experimental confirmation. Relevant work is now underway in our research team and will be published in the future.

**Figure 13:**
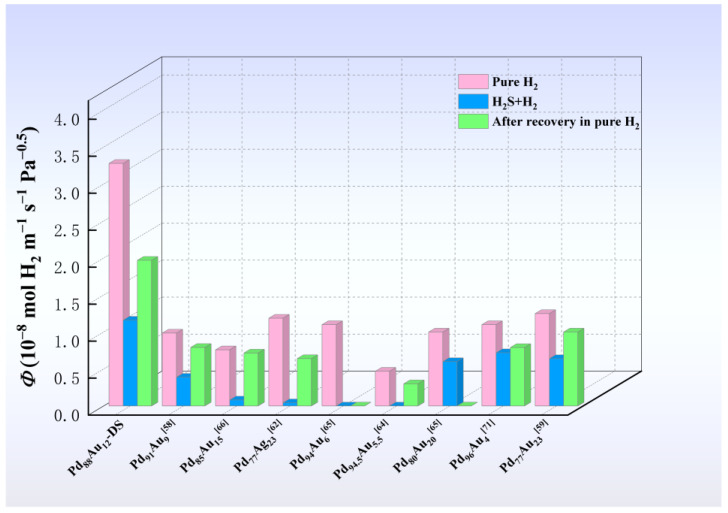
Comparison of hydrogen permeability through the composite membranes measured in pure H_2_, H_2_/H_2_S mixture (673 K), and after steady flux recovery in pure H_2_.

Finally, the characteristics of these sandwich-like composite membranes are discussed by comparing and analysing similar alloy membranes reported in the literature [62,63,64,65,66,67,71], as shown in Figure 13. Firstly, compared with other self-supported bulk Pd-based alloys (e.g., Pd–Ag/ZrO_2_ [72]), the membrane examined here exhibited not only a higher pure H_2_ permeability but also lower manufacturing costs due to the unique sandwich structure of the PdAu-coated membranes. For example, a 0.5 mm thick Pd_77_Au_23_ membrane would cost approximately USD 475,000 per m^2^, while DS-Nb_15_Ti_55_Fe_30_ (0.7 mm thick) coated with 200 nm of Pd_88_Au_12_ only costs around USD 160 per m^2^ (pure Pd-coated about USD 165 per m^2^). The price differential between the use of pure Pd and Pd_88_Au_12_-coated is not substantial; however, the resistance to sulphide of the membrane is markedly enhanced.

Secondly, due to the high permeability of the base-metal layer in the middle, these composite membranes demonstrate higher permeability than other bulk Pd-based alloys in both H_2_ and mixed H_2_/H_2_S gas environments. This characteristic facilitates achieving higher permeability, even with higher inhibition from the presence of H_2_S. For example, the Pd_94_Au_6_ composite membrane exhibits lower hydrogen permeance inhibition compared to the newly developed sample in this article. However, the hydrogen permeability of the former is lower than that of the latter. If the components are designed reasonably, the PdAu-coated DS-Nb_15_Ti_55_Fe_30_ composite membranes are more likely to achieve better H_2_ permeability combined with good sulfur resistance and reduced membrane costs.

Lastly, these sandwich-like membranes could achieve infinite hydrogen selectivity in hydrogen separation and purification, as long as the Nb matrix in the middle does not undergo HE fracture. In this case, even if the PdAu catalytic layer on the surface is damaged, the selectivity remains unaffected. In comparison, the selectivity of single-layer self-supported Pd-based films will sharply decrease or even disappear after damage. Similar cases may also occur in Pd-based multilayer composite membranes composed of a porous matrix, such as porous stainless-steel supports and porous ceramics [73].

In summary, this article proposes a new method of modifying the composition of the membrane catalytic layer to tailor hydrogen permeability, maximise H_2_ flux, and inhibit sulfur poisoning, which is extremely important for the subsequent design of group 5B alloy membranes.

## 4. Conclusions

The dural Nb-TiFe regular eutectic structure can be prepared by directional solidification technology at growth rates below 1 mm h^−1^, thereby improving the pure H_2_ permeance. This further substantiates that Nb rods arranged continuously along the direction of membrane permeation are beneficial for hydrogen permeation. Additionally, a decline in the permeability of Pd-coated membranes was observed upon exposure to H_2_S, with higher H_2_S concentrations resulting in greater permeance loss. This poisoning behaviour can be effectively improved and alleviated by using a modified surface catalytic layer (i.e., replacing pure Pd with PdAu). Notably, a Pd_88_Au_12_-containing membrane showed higher permeability, permeance recovery, and better resistance to bulk sulfidation than a pure Pd-coated membrane.

In the Pd_88−x_Au_12+x_-coated alloy series, lower Au content resulted in higher pure H_2_ permeability, whilst recovery rate after the H_2_S tests was positively correlated with Au content. Therefore, the composition selection of the outermost PdAu catalytic layer was a compromise, and the Pd_88−x_Au_12+x_ (0 ≤ x ≤ 6) coated membrane showed relatively satisfactory properties with respect to hydrogen permeation in the presence of H_2_S. Multi-component gas testing (involving three or more types, selected according to the U.S. DOE guidelines) is underway to evaluate the chemical tolerance of these synthesised membranes under similar conditions, such as test temperature and pressure difference.

## Figures and Tables

**Table 1:** Chemical atomic composition and constituting phases of the synthesised samples.

Sample	Composition (at. %)						Constituting Phases	Orientation Relationship
	Nominal			Measured				
	Nb	Ti	Fe	Nb	Ti	Fe		
AC-Nb_15_Ti_55_Fe_30_ *	Bal.	55	30	Bal.	55.1	29.8	bcc-Nb, TiFe	[110]bcc-Nb/[110]TiFe [001]bcc-Nb/[001]TiFe
DS-Nb_15_Ti_55_Fe_30_ *	Bal.	55	30	Bal.	54.2	29.5	bcc-Nb, TiFe	None

* The symbols AC and DS represent the abbreviation for as-cast and directional solidification, respectively.

**Table 2:** The numbering of Pd- and/or Pd_88_Au_12_-coated Nb_15_Ti_55_Fe_30_ alloys and their hydrogen permeation performance when exposed to different H_2_S concentrations.

Nos.	Sample	Catalytic Layer (at. %)	H_2_S Concentration (ppm)	Temperature (K)	Hydrogen Permeability, *Φ* (10^−8^ mol H_2_ m^−1^ s^−1^ Pa^−0.5^)
1#	AC-Nb_15_Ti_55_Fe_30_	Pd	0	673	2.65
2#	DS-Nb_15_Ti_55_Fe_30_	Pd	0	673	3.71
			1	673	3.38
			60	673	2.81
			100	673	0.51
3#	DS-Nb_15_Ti_55_Fe_30_	Pd_88_Au_12_	0	673	3.26
			100	673	1.16

**Table 3:** Comparison of the literature results of leading Pd-alloy membranes exposed to a H_2_/H_2_S mixture and the ones presented in this work.

Reference	Membrane Composition	Fabrication Technique *	Temperature (K)/Time (h) of Exposure	Feed Composition (H_2_S/H_2_)	Decline Rate of Permeability (%)	H_2_ Recovery (%)	Sulfide Species
McKinley [44]	Pure Pd/Prous stainless steel	MS	623/144	4.7 ppm H_2_S/H_2_	70	100	No sulfide formation
Mundschau [45]	Pure Pd	Pd foil	593/120	20 ppm H_2_S/H_2_	72	—	Pd_4_S
Braun [60]	Pure Pd	ELP	623/24	100 ppm H_2_S/H_2_	90	8	Pd_4_S
Peters [61]	Pure Pd	MS	723/1	20 ppm H_2_S/10%	92	85	—
Mundschau [45]	Pd_75_Ag_25_	-	593/72	10 ppm H_2_S/H_2_	84	—	Pd_4_S and Ag_5_Pd_10_S_5_
Peters [62]	Pd_77_Ag_23_	MS	723/1	20 ppm H_2_S in 90% H_2_ in N_2_	95	50	Pd_4_S and Ag_5_Pd_10_S_5_
Braun [60]	Pd_90_Ag_10_	ELP	673/24	100 ppm H_2_S/H_2_	75	32	Pd_4_S and Ag_5_Pd_10_S_5_
Kulprathipanja [63]	Pd_27_Cu_73_	ELP	723 K/10	250 ppm H_2_S in N_2_/H_2_	90	30	—
Kulprathipanja [57]	Pd_68_Cu_32_	MS	723/145	100 ppm H_2_S in N_2_/H_2_	40	66	CuS
Chen [64]	Pd-8 wt.% Au	ELP	673/5	54.8 ppm H_2_S/H_2_	85	67	No sulfide formation
McKinley [44]	Pd_60_Au_40_	MS	623/192	4 ppm H_2_S/H_2_	21	—	—
Lewis [59]	Pd_77_Au_23_	ELP	773/70	20 ppm H_2_S/WGS	29	97	No sulfide formation
Gade [65]	Pd_80_Au_20_	CW	673/100	20 ppm H_2_S/WGS	42	—	Pd_4_S and Pd_2.8_S
Peters [66]	Pd_85_Au_15_	MS	723/1	100 ppm H_2_S/H_2_	90	88	—
Lewis [67]	Pd_90_Au_10_	MS	773/48	5 ppm H_2_S/H_2_	60	75	—
This work	Pd/DS-Nb_15_Ti_55_Fe_30_/Pd	MS	673/8	100 ppm H_2_S/H_2_	86.5	0.3	Pd_4_S, Pd_2.8_S and Pd_16_S_7_
This work	Pd_86_Au_14_/DS-Nb_15_Ti_55_Fe_30_/Pd_86_Au_14_	MS	673/8	100 ppm H_2_S/H_2_	63.2	58.3	No sulfide formation

* ELP, MS and CW are short for electroless plating, magnetron sputtering and cold-working, respectively.

## Data Availability

The original contributions presented in the study are included in the article, further inquiries can be directed to the corresponding author.
